# Are You Really Paranoid, Just Because They Are After You? Exploring the Underlying Sensitization Processes of Intersectional Discrimination on Everyday-Life Paranoia

**DOI:** 10.32872/cpe.19263

**Published:** 2026-05-29

**Authors:** Björn Schlier, Felix Strakeljahn, Christoph Kahl, Katharina Winkler

**Affiliations:** 1 Department of Clinical Child and Adolescent Psychology and Psychotherapy, University of Wuppertal, Wuppertal, Germany; 2 Department of Clinical Psychology and Psychotherapy, University of Hamburg, Hamburg, Germany; Philipps-University of Marburg, Marburg, Germany

**Keywords:** discrimination, minority, intersectionality, paranoia, psychosis, ambulatory assessment

## Abstract

**Background:**

Elevated paranoia levels have been found in discriminated, minoritized groups. Social-cognitive models of paranoia posit that experiences of discrimination strengthen negative core beliefs, which in turn foster clinical paranoia. In contrast, the healthy cultural mistrust hypothesis proposes that elevated paranoia in minoritized groups reflects an adaptive response to increased exploitation/discrimination. To explore whether elevated paranoia in discriminated groups can be fully understood within clinical models, we tested whether a history of discrimination amplifies the association between everyday-life stressors and subsequent state-paranoia and whether this moderation effect remains when controlling for established cognitive risk factors for paranoia (i.e., dysfunctional beliefs).

**Method:**

A general population sample (*n* = 108) answered a baseline self-report questionnaire of lifetime discrimination experiences (LDE) and core beliefs about oneself and others. Next, they reported state-paranoia, negative affect, and exclusion experiences in 2.5h intervals for seven days of ambulatory assessment. LDE and core-beliefs were tested as concurrent moderators for the associations between state-paranoia and putative triggers (negative-affect, exclusion) using multilevel regression.

**Results:**

More LDE amplified the association between paranoia, negative affect, and exclusion experiences. When controlling for the moderation effects of negative beliefs, effect sizes for the LDE moderation were lower but largely remained significant.

**Conclusions:**

Increased state-paranoia in the daily lives of people with discrimination experiences can be partially, but not fully, explained by clinical, cognitive risk factors. Consequently, healthy cultural mistrust can be considered an additional contributing factor to higher mistrust/paranoia in minoritized groups. Future in-depth research needs to disentangle mechanisms of emerging paranoia and adaptive mistrust in minoritized groups.

## Highlights

- Lifetime discrimination experiences (LDE) correlate strongest with lifetime paranoia among psychotic symptoms.
- LDE amplifies cross-sectional associations between state-paranoia, –negative affect, and –exclusion.
- LDE amplifies time-lagged associations from exclusion to paranoia to negative affect.
- LDE effects remain when controlling for clinical risk factors, implying healthy cultural mistrust.

Various etiological models highlight the crucial role of social adversity in the emergence of clinical levels of psychosis and specific symptoms such as paranoia (e.g., Jaya et al., 2016; van Os et al., 2010). Cognitive theories (Beck et al., 2011; Freeman et al., 2002; Garety et al., 2001; Kesting & Lincoln, 2013; Morrison, 2001) suggest that paranoia results from such experiences via cognitive biases rooted in dysfunctional, i.e., more negative and/or less positive, beliefs about oneself and others. The *Social Defeat Hypothesis* (Selten et al., 2013) proposes that the underlying common factor of established risk factors for psychotic symptoms, including paranoia, such as migration, urban upbringing, or even illicit drug use, is experiencing social defeat or exclusion. Specifically, chronic social defeat/exclusion sensitizes the mesolimbic dopamine system, increasing the risk that subsequent stressors elicit excessive stress responses and thus the emergence of psychotic symptoms. Such frameworks allow us to explain how stressors found to be linked to psychotic symptoms may lead to the emergence of symptom-states in some individuals but not in others. According to the *affective pathway model to psychosis* (Myin-Germeys & Van Os, 2007), fluctuations in negative affect that follow a stressful event constitute a common stressor, with multiple ambulatory assessment studies linking negative affect levels to subsequent emergence and/or increase in psychotic symptoms in daily life (Krkovic et al., 2020; Ludwig et al., 2020). In recent years, some experimental (e.g., Lincoln et al., 2018) and ambulatory assessment studies (e.g., Monsonet et al., 2022; Schlier et al., 2018) have shown that momentary experiences of social defeat or social exclusion can also serve as a situational trigger of positive symptoms such as hallucinations or paranoia. This tentatively suggests that not only a history of social defeat/exclusion (that affects vulnerability), but also current momentary experiences of exclusion (as a specific trigger) constitute relevant risk factors for psychosis. Which symptoms are directly triggered by social defeat/exclusion, however, is still an open question (Shovestul et al., 2023).

Some researchers have tried to focus on individual symptoms rather than broad diagnostic or syndrome categories to reveal risk factors and/or stressors that are specific to the emergence of paranoia, hallucinations, or other psychotic symptoms (Bentall et al., 2014). Such a symptom-specific approach may not just expand our understanding of the aetiology of heterogeneous expressions of psychosis but also guide case formulation via the identification of an individual’s most effective treatment targets for psychotherapeutic interventions. For paranoia, one such putatively specific risk factor is discrimination. A review on the topic of the association between discrimination experience and psychosis showed that a substantial number of studies found a specific association between discrimination experience and paranoia, specifically in at-risk and population samples (Pearce et al., 2019). Discussing these finding, Pearce and colleagues (2019) drew a parallel to findings of more pronounced negative core beliefs about the self and others in minority samples as well as “intrinsic social inequalities that underpin discrimination” that might lead to experiences of social defeat, proposing discrimination to be a vulnerability factor for paranoia that could be explained within the framework of existing clinical models.

At the same time, disparities in the U.S.-American health-care system, particularly increased rates of misdiagnosis of psychosis in ethnic minorities and their under-utilization of the mental health care system, have been viewed under the assumption of *healthy cultural mistrust* (Whaley, 2001b). Cultural mistrust describes a response style that results from experiences of discrimination/oppression, which informs a person’s attitudes about the majority group in society, leading to a functional apprehensiveness in subsequent interactions with members of that group (Whaley, 2001a). Thus, the healthy cultural mistrust hypothesis suggests that what is phenomenologically perceived as paranoia in minoritized groups could reflect a healthy, adaptive response to discrimination (Whaley, 2001b). From this perspective, paranoid responses rooted in a history of discriminatory experiences could be etiologically distinct from paranoid responses rooted in the aforementioned clinically relevant social-cognitive mechanisms. At worst, they could constitute a phenomenological expression of a distinct form of distress, e.g., depression clad in contextually-evoked suspiciousness, that is diagnostically misclassified as paranoia.

Pearce and colleagues (2019) found that evidence on the connection between discrimination and paranoia primarily stems from cross-sectional studies, limiting our understanding of the underlying mechanisms connecting the two. Utilizing a large multi-cultural dataset, Kingston et al. (2023) highlighted potential differences in paranoia as reported by minoritized groups vs. the majority. They showed that paranoia levels were higher in people from minoritized groups and increased with more intersectionality: With each additional minoritized group people identified with, paranoia levels were higher. Intriguingly, various associations between established social-cognitive risk factors found to correlate with paranoia in majority groups (i.e., positive core beliefs about the self, about others, and low social rank beliefs as an indicator of habitual social defeat) did not show the same association in (intersectionally) minoritized groups. This tentatively suggests that there is a distinct underlying mechanism in minoritized groups, such as healthy cultural mistrust.

To further investigate these putative etiological differences, the current study re-analyses an existing ambulatory assessment dataset to investigate the influence of lifetime experiences of discrimination on current experiences of paranoia and their association with established momentary triggers of paranoid states. In this (due to the authors’ prior knowledge of the data) not pre-registered secondary analysis, we aim to explore to what extent and by what mechanism the lifetime experiences of discrimination sensitize people to the experience of paranoia. In a first step, we explore to what degree the lifetime discrimination experience (LDE) attributable to belonging to one or more minoritized groups, i.e., increasing levels of intersectional LDE, correlate with lifetime experiences of paranoia. Additionally, we compare this correlation with associations of LDE and the lifetime experience of other psychotic symptoms. Further, we explore to what extent LDE covaries with changes in core beliefs about oneself and other people. In the second step, we test whether LDE affects the daily life experience of aversive social situations (i.e., social exclusion), negative affect, and paranoid states. To this end, we first test whether more LDE covaries with higher person-average levels of social exclusion, negative affect, and paranoid states in daily life. Next, we test whether LDE modulates the effects of state-social exclusion, -negative affect, and -paranoia exerted on each other. Finally, we investigate whether any such modulating effects can be explained by differences in core beliefs associated with discrimination.

## Method

### Design and Procedure

The data for this study was derived from a larger study on social antecedents of psychosis symptoms. It consisted of a baseline assessment followed by a seven-day ambulatory assessment period. Participants provided informed consent and then completed a 20-to-30-minute questionnaire battery comprising self-report measures on lifetime psychotic experience, core beliefs about the self, minority status, discrimination, and other interpersonal and aversive experiences. The questionnaire was presented on the survey platform Questback EFS.

Next, an ambulatory assessment was conducted using the app movisensXS. The ambulatory assessment started immediately after the baseline assessment and continued for seven days. Participants either received an Android smartphone with the app movisensXS preinstalled (*n* = 12) or installed the app on their smartphones (*n* = 96). Participants received the first assessment prompt during the introductory session to ensure there were no technical problems with the app and to answer any questions on handling the app. On the day of the baseline assessment, participants received one to five prompts, depending on the time of the first alarm. On the following six days, participants received five alarms per day, presented between 10 am and 9 pm in 2.5-hour intervals (+/-15min). Thus, the total number of questionnaires presented during the ambulatory assessment phase ranged from 31 to 35. The completion of an assessment questionnaire took approximately five minutes. If participants were unable to answer the questionnaire immediately after the prompt, it could be postponed by an additional ten minutes.

After seven days, participants returned to our lab for debriefing, to return the smartphone they received for the study, and to provide brief feedback on any problems or unusual events during the assessment.

### Materials

#### Baseline Assessment

At baseline, lifetime experience of discrimination (LDE) was assessed with a modified version of the discrimination assessment from the NEMESIS study (Janssen et al., 2003), which has been used in this form multiple times in large-scale surveys (Jaya et al., 2016; Kingston et al., 2023). Using a yes/no answer format, participants first indicated whether they belonged to any of five minority group categories, i.e., ethnicity, religion, sexual orientation/identity, disability, or physical differences (such as obesity or visible scars). Following this, they were presented with two to seven additional yes/no questions asking whether they experienced discrimination due to being a member of any of the previously mentioned minority groups (individual questions only presented when participants indicated they were a member of the corresponding minority) or because of their gender or age (questions presented to every participant). A total score of all positively answered discrimination questions, ranging from 0 to 7, was calculated as an indicator of the extent of intersectional LDE. Higher values indicate LDE due to more coinciding forms of discrimination.

Lifetime prevalence of psychotic experiences was assessed with the validated German version of the Community Assessment of Psychic Experience (CAPE). This 42-item self-report questionnaire measures psychotic experiences in nine symptom categories that can further be summarized into three dimensions (Schlier et al., 2015): The dimension positive symptoms (20 items, e.g., “Do you ever feel as if people seem to drop hints about you or say things with a double meaning?”), consisted of the symptoms paranoid beliefs (five items), bizarre experiences (seven items), hallucinations (four items), grandiosity (two items), and magical thinking (two items); Negative symptoms (14 items, e.g., “Do you ever feel that you are not a very animated person?”), consisted of the symptoms amotivation (seven items), anhedonia (three items), and social withdrawal (four items). Finally, depressive symptoms (eight items, e.g., “Do you ever feel sad?”) constituted a third dimension with no subfactors. All items are answered on four-point Likert-scales (1 = “never”, 2 = “sometimes”, 3 = “often”, 4 = “nearly always”). The German version of the CAPE has shown good reliability and validity across population and patient samples (Schlier et al., 2015). Mean scores for symptom dimensions and individual symptom factors were calculated.

Core beliefs about oneself and others were assessed with the Brief Core Schema Scale (BCSS; Fowler et al., 2006), a 24-item questionnaire assessing cognitive-schemas particularly relevant in psychosis. Specifically, the four dimensions negative-self-beliefs (e.g., “I am worthless”), positive-self-beliefs (e.g., “I am good”), negative-other-beliefs (e.g., “other people are nasty”), and positive-other-beliefs (e.g., “other people are supportive”) are assessed with six items each. Participants rated the items on five-point Likert scales (1 = “no, do not believe it”; 5 = “yes, fully believe it”). The BCSS has shown good validity and high internal consistency across all four dimensions (Fowler et al., 2006), Cronbach’s α ranged from .78 to .88, and its German translation has been used in multiple population studies (e.g., Kingston et al., 2023). Mean scores for each of the four core-schema dimensions were calculated for this study.

Additionally, we assessed habitually experienced social exclusion as an indicator of baseline social defeat, using the three-item inclusion-subscale from the *Social Comparison Scale* (SCS; Allan & Gilbert, 1995). The SCS measures different aspects of social comparisons with others (i.e., social rank, social attractiveness, and inclusion/exclusion). All items are ten-point semantic differentials between adjective pairs. The three-item inclusion-subscale focuses on the subjective feeling of being accepted vs. excluded by others (e.g., “In relationship to others I feel: left out – accepted“). At baseline, participants were asked to indicate how they “usually” feel in relationships with others. Social defeat assessments vary and ideally include multiple indicators – often encompassing feelings of exclusion, defeatist beliefs and self-reported discrimination or minority status (e.g., Shovestul et al., 2023). Nevertheless, prolonged/repeated feelings of exclusion are often described as core features of chronic social defeat (e.g., Selten et al., 2016). We calculated a reversed mean-score as an indicator of exclusion, with higher values indicating more intense feelings of social defeat.

#### Ambulatory Assessment

The ambulatory assessment included short self-report measures on current negative affect, psychotic experiences, and social interactions. All 32 items (2-6 per variable, see Supplementary Materials, Table S1) referred to the time period since the last prompt, except for negative affect, which referred to the current moment. For the analyses in this study, negative affect, paranoia, and social exclusion assessments are used.

To assess current negative affect, participants were asked to rate their current feelings of anxiety, anger, sadness, and shame based on a list of four adjectives (e.g., anxiety: “anxious, fearful, afraid, worried”). Participants rated each item on an 11-point Likert scale (0 = “does not apply to me at all”, 10 = “strongly applies to me”). These items have been used in multiple ambulatory assessment studies (e.g., Krkovic et al., 2018, 2020). A negative affect mean score for each assessment was calculated.

State paranoid ideation was assessed using the five-item Brief State Paranoia Checklist (Schlier et al., 2016), which was developed as an ambulatory assessment ready, change-sensitive version of the Paranoia Checklist (Freeman et al., 2005). It includes items on milder social-evaluative concerns (e.g., “People laughed at me.”) to more prototypically clinical paranoia (e.g., “My actions/thoughts might have been controlled by others”). Items were answered on 11-point Likert scales (0 = “does not apply to me at all”, 10 = “strongly applies to me”). The five-item version of the Paranoia Checklist has shown sufficient reliability with a Cronbach’s α of .83, within-person variability, and validity in its validation samples (Schlier et al., 2016).

State social exclusion was again assessed with the three-item subscale “group fit” from the *SCS* (Allan & Gilbert, 1995). The German translation of the scale was adopted from a previous ambulatory assessment study (Schlier et al., 2018). Participants were asked how they perceived themselves in relation to others since the last prompt. A reversed mean-score was calculated as an indicator of state social exclusion (i.e., higher values indicate more social exclusion).

### Participants

Participants were recruited at the University of Hamburg via advertisement on Campus and in the digital participant recruitment database of the department of psychology. Demographic characteristics of the 108 participants are summarized in Table 1. The participants were young (mean age: 24.24 years), consisted mostly of university students (95.37%) with up to 1,000€ of monthly income (76.85%), and predominantly identified as women (77.78%). About half of the sample reported they have experienced one or more types of discrimination (52.78%), with the most prevalent forms being discrimination due to gender (33.33%), age (25.93%), visible physical differences (e.g., obesity or scars: 8.33%), and ethnicity/religion (6.48%, respectively).

**Table 1:** Demographic Information of the Study Sample

Demographic Variable	*M* or %	*SD*	*Range*
**Age**	24.24	5.68	18-43
Gender			
Man	22.22%	–	–
Woman	77.78%	–	–
Other	–	–	–
No Answer	–	–	–
Current occupation			
Student	95.37%	–	–
Employed	3.70%	–	–
Self-employed	0.92%	–	–
Monthly income			
up to 500€	40.74%	–	–
500€ to 1,000€	36.11%	–	–
1,000€ to 1,500€	13.89%	–	–
1,500€ to 2,000€	5.56%	–	–
more than 2,000€	3.70%	–	–
Minority status due to…			
…ethnic minority	12.96%	–	–
…sexual orientation/identity	6.48%	–	–
…religious beliefs	7.41%	–	–
…visual physical difference	12.00%	–	–
Discrimination Experience			
Reporting any type of lifetime discrimination	52.78%	–	–
Experience of discrimination due to…			
…Age	25.93%	–	–
…Gender	33.33%	–	–
…Ethnicity	6.48%	–	–
…Sexual orientation/identity	3.70%	–	–
…Religious beliefs	6.48%	–	–
…Visual physical difference	8.33%	–	–
Number of lifetime discrimination types experienced			
All participants	0.84	0.98	0-4
…Participants reporting any discrimination (*n* = 57)	1.60	0.78	1-4

*Note.* *M* = Mean; *SD* = standard deviation.

The study was approved by the ethics committee of the University of Hamburg. All participants provided written informed consent prior to participation in accordance with the Declaration of Helsinki.

### Data Analysis

To test for and compare the association between LDE and different types of lifetime psychotic experiences, we calculated Spearman correlation tests between LDE and CAPE symptom dimensions/factors. Additionally, Spearman correlation tests were calculated between LDE and BCSS scores to identify cognitive changes that potentially underlie sensitization processes.

Next, we tested for the effect of LDE on state negative affect, paranoia, and social exclusion by calculating random-intercept, fixed-slope multilevel models with LDE as level-two predictors and either state negative affect, state paranoia, or state social exclusion as outcome. Further, to test for the sensitization effects of LDE, we calculated multilevel models with one of the state variables, negative affect, paranoia, or social exclusion as the dependent variable and another of the three state variables, as well as the intersectional LDE index, as independent variables. Both cross-sectional and time-lagged model variations were calculated for each of these combinations. Time-lagged models were built with a one-assessment-interval lag, i.e., 2.5 hours, and also controlled for the dependent variable scores at the prior time-point. Interaction effects between the respective independent variable x LDE were tested.

Finally, we tested for potential sensitization effects due to baseline core schema levels that putatively vary as a function of LDE within a clinical vulnerability perspective on paranoia. We entered interactions with any risk factor significantly associated with LDE in the initial correlation tests as parallel moderators to the multilevel models. In these models, we tested for a combination of significant risk factor x independent variable interaction and no-longer-significant LDE x independent variable interactions. The significance level for each individual test was *p* < .05.

## Results

All participants fully answered all questionnaires at baseline. In the ambulatory assessment, total compliance was 86.14%, with 57 participants answering more than 90% of their questionnaires, 39 answering 70%-90%, nine answering 50%-70%, and three answering between 40%-50% of their questionnaires.

### Association of LDE With Lifetime Psychotic Experiences and Risk Factors

The results of all correlation tests between LDE and lifetime psychotic symptoms are summarized in Table 2. There were significant associations with positive symptom scores, paranoia scores, bizarre experiences scores, amotivation scores, and depression scores. As expected, the highest correlation was between LDE and lifetime paranoia, *r* = .48. Lifetime positive symptoms also showed a significant, yet only medium association, *r* = .39. The majority of the remaining correlations amounted to non-significant, small to medium effect sizes, 0.11 ≤ *r* ≤ 26, except for grandiosity, *r* = .07, and social withdrawal, *r* = -.02. Post-hoc *z*-tests for differences between correlations controlling for the association between the symptom variable and paranoia (added as suggested during revision) showed paranoia to correlate significantly stronger with LDE than all other symptoms and symptom-dimensions, except for the positive symptom sum-score, *z* = 1.60, *p* = .055.

**Table 2:** Correlation Test Results Between Lifetime Discrimination Experience (LDE) and Lifetime Psychotic Symptoms, Social Defeat, and Self and Other Beliefs at Baseline

Symptom/Risk Factor	Descriptive Values			Correlation with LDE			Test for difference with correlation LDE-Paranoia	
	*M*	*SD*	*Range*	*r*	*t*(106)	*p*	*z*	*p*
Positive symptoms	1.40	0.20	1.05-2.10	**0.39**	**4.37**	**< .001**	1.60	.055
Paranoia	1.79	0.37	1.00-2.80	**0.48**	**5.67**	**< .001**	–	–
Bizarre experiences	1.19	0.23	1.00-2.00	**0.23**	**2.48**	**.015**	**2.62**	**.004**
Hallucinations	1.05	0.17	1.00-2.50	0.13	1.34	.183	**3.19**	**.001**
Grandiosity	1.87	0.61	1.00-3.50	0.07	0.70	.488	**3.63**	**< .001**
Magical Thinking	1.41	0.45	1.00-3.00	0.15	1.56	.122	**2.98**	**.001**
Negative symptoms	1.91	0.34	1.21-2.79	0.18	1.91	.059	**2.79**	**.003**
Amotivation	1.96	0.42	1.00-3.14	**0.25**	**2.62**	**.010**	**2.22**	**.013**
Anhedonia	1.65	0.57	1.00-3.33	0.11	1.10	.273	**3.08**	**.001**
Social withdrawal	2.03	0.44	1.25-3.25	-0.02	-0.23	.819	**4.18**	**< .001**
**Depression**	1.98	0.37	1.13-3.63	**0.26**	**2.72**	**.008**	**2.25**	**.012**
**Baseline social defeat**	14.49	5.41	3.00-27.00	**0.24**	**2.55**	**.012**	–	–
**Negative self-beliefs**	3.22	2.95	0.00-18.00	**0.22**	**2.32**	**.022**	–	–
**Positive self-beliefs**	14.61	5.10	2.00-24.00	-0.03	-0.34	.735	–	–
**Negative other-beliefs**	3.77	3.18	0.00-12.00	**0.29**	**3.14**	**.002**	–	–
**Positive other-beliefs**	13.02	4.38	3.00-24.00	-0.06	-0.58	.565	–	–

*Note.* Symptom scores range: 1 to 4; self/other belief scores range: 0 to 24; social defeat score range: 3 to 33. Significant correlations (*p* < .05) are printed in bold.

Regarding the association between LDE and core beliefs, there were significant associations with habitual social defeat, *r* = .24, negative-self-beliefs, *r* = .22, and negative-other-beliefs, *r* = .29, but not with positive-self-beliefs, *r* = -.03, or positive-other-beliefs, *r* = -.06 (see Table 2).

### The Effects of LDE on Everyday Life Experiences

Multilevel models using LDE as a level two predictor showed that people with more LDE reported higher levels of negative affect, β = 0.27, *b* = 0.16, *SE* = 0.06, *t* = 2.74, *p* = .007, and paranoia, β = 0.46, *b* = 0.22, *SE* = 0.04, *t* = 5.10, *p* < .001, but not social exclusion in everyday life, β = 0.11, *b* = 0.16, *SE* = 0.14, *t* = 1.13, *p* = .263.

Table 3 provides an overview of all the moderation effects of LDE. As can be seen, the effect of social exclusion on paranoia is amplified by LDE in both the cross-sectional, β = 0.24, and time-lagged regression, β = 0.13. Further, there are significant moderation effects in the cross-sectional models for negative affect on paranoia, β = 0.35, and social exclusion, β = 0.10, plus paranoia on social exclusion, β = 0.10, with no corresponding effects in time-lagged analyses. Finally, the effect of paranoia on negative affect was significantly moderated in time-lagged analysis only, β = 0.08.

**Table 3:** Discrimination Experiences as Moderator for the Associations Between Everyday-Life Social Exclusion, Negative Affect, and Paranoia

Outcome	Predictor	Models Including Predictor and Discrimination										Models Controlling for Core-Schemas and Social Defeat					
		Main Effect Predictor					Discrimination x Predictor					Discrimination x Predictor					Other Mod.
		β	*b*	*SE*	*t*	*p* ^a^	β	*b*	*SE*	*t*	*p* ^a^	β	*b*	*SE*	*t*	*p* ^a^	Range β
Cross-Sectional Models																	
Paranoia	Social excl.	**0.22**	**0.07**	**0.01**	**6.41**	**< .001**	**0.24**	**0.04**	**0.01**	**6.31**	**< .001**	**0.15**	**0.03**	**0.01**	**3.63**	**< .001**	-0.14; 0.17
Social excl.	Paranoia	**0.25**	**0.45**	**0.06**	**7.19**	**< .001**	**0.10**	**0.09**	**0.03**	**2.76**	**.006**	**0.09**	**0.08**	**0.04**	**2.20**	**.028**	-0.05; 0.11
Paranoia	Neg. affect	**0.27**	**0.16**	**0.02**	**10.38**	**< .001**	**0.35**	**0.12**	**0.01**	**11.62**	**< .001**	**0.26**	**0.09**	**0.01**	**8.08**	**< .001**	-0.13; 0.29
Neg. affect	Paranoia	**0.48**	**0.57**	**0.04**	**14.45**	**< .001**	0.03	0.02	0.02	0.88	.378	0.00	0.00	0.02	< 0.01	0.999	-0.03; 0.10
Social excl.	Neg. affect	**0.34**	**0.42**	**0.04**	**12.32**	**< .001**	**0.10**	**0.07**	**0.02**	**3.08**	**.002**	0.06	0.05	0.03	1.80	0.072	-0.01; 0.22
Neg. affect	Social excl.	**0.46**	**0.22**	**0.02**	**13.86**	**< .001**	0.00	0.00	0.01	0.07	.943	**-0.08**	**-0.02**	**0.01**	**-2.04**	**.041**	-0.08; 0.32
Lagged Models																	
Paranoia	Social excl.	-0.02	-0.01	0.01	-0.63	.529	**0.13**	**0.02**	**0.01**	**3.16**	**.002**	**0.12**	**0.02**	**0.01**	**2.65**	**.008**	-0.09; 0.11
Social excl.	Paranoia	0.01	0.02	0.08	0.28	.780	0.05	0.04	0.04	1.27	.206	0.02	0.02	0.04	0.51	.612	-0.03; 0.05
Paranoia	Neg. affect	0.03	0.02	0.02	0.96	.340	-0.02	-0.01	0.01	-0.67	.500	-0.06	-0.02	0.01	-1.61	.107	0.03; 0.06
Neg. affect	Paranoia	-0.03	-0.04	0.05	-0.83	.405	**0.08**	**0.05**	**0.02**	**2.08**	**.037**	0.04	0.03	0.03	1.01	.315	-0.08; 0.13
Social excl.	Neg. affect	-0.01	-0.01	0.04	-0.26	.489	0.05	0.03	0.03	1.33	.182	0.01	0.01	0.03	0.35	.729	-0.02; 0.07
Neg. affect	Social excl.	0.02	0.01	0.02	0.45	.654	0.07	0.02	0.01	1.80	.072	0.06	0.01	0.01	1.31	.190	-0.01; 0.13

*Note*. Neg. affect = negative affect; Social excl. = social exclusion. All models are random-intercept, fixed slope multilevel-regressions with predictor, discrimination index, and predictor x discrimination index as independent variables; Controlled models (third column-cluster) also include grand-mean-centered BCSS negative other-beliefs scores, BCSS negative self-beliefs scores, and SCS social rank beliefs and corresponding interaction effects with the predictor. Range β provides an overview of the standardized effects of the moderations entered as control variables. Time-lagged models are additionally controlled for the effect of the dependent variable scores from the previous assessment.

^a^*p*-values calculated with Satterthwaite approximation, with significant effects (*p* < .05) printed in bold.

### Comparison of Moderation Effects of LDE vs. Negative Core and Social Beliefs

The results of the moderation models testing the simultaneous effect of LDE and clinical cognitive risk factors from baseline (negative-other-beliefs, negative-self-beliefs, and social defeat) are summarized in Table 3 (right columns). All previously significant LDE moderation effects remained significant, except for the paranoia x LDE effect on subsequent negative affect.

Overall, standardized moderation effect sizes for LDE decreased when controlling for the clinical risk factors. However, in the models with a significant LDE-moderation effect, effect sizes of the concurrently tested moderations (see rightmost column of Table 3 for an overview and Table 4 for a detailed list of other moderation effects) were mostly within the same range. Notably, the controlled cross-sectional effect of social-exclusion x LDE, β = 0.15, was only marginally smaller than the highest other interaction effect for social exclusion x negative-other-beliefs, β = 0.17. For the time-lagged association between social exclusion and subsequent paranoia, the LDE moderation effect was highest, β = 0.13, albeit only marginally higher than the second-highest moderation effect, negative-self-beliefs: β = 0.11.

**Table 4:** Discrimination Experiences as Moderator for the Associations Between Social Exclusion, Negative Affect, and Paranoia: Overview of all Tested Moderators

Outcome	Predictor	Single moderator	Model with all moderation effects included											
		Predictor x Discrimination	Predictor x Discrimination			Predictor x Negative Other-Beliefs			Predictor x Negative Self-Beliefs			Predictor x Baseline Social Defeat		
		β	β	*t*	*p* ^a^	β	*t*	*p* ^a^	β	*t*	*p* ^a^	β	*t*	*p* ^a^
Cross-Sectional Models														
Paranoia	Social excl.	**0.24**	**0.15**	**3.63**	**< .001**	**0.17**	**3.78**	**< .001**	**-0.14**	**-3.13**	**.002**	**0.12**	**2.64**	**.008**
Social excl.	Paranoia	**0.10**	**0.09**	**2.20**	**.028**	-0.05	-1.34	.179	**-0.08**	**-2.12**	**.034**	**0.11**	**3.19**	**.001**
Paranoia	Neg. affect	**0.35**	**0.26**	**8.08**	**< .001**	**0.29**	**10.04**	**<.001**	0.00	-0.01	.994	**-0.13**	**-3.97**	**< .001**
Neg. affect	Paranoia	0.03	0.00	< 0.01	.999	**0.10**	**3.01**	**.003**	0.06	1.60	.109	-0.03	-0.99	.322
Social excl.	Neg. affect	**0.10**	0.06	1.80	.072	-0.01	-0.46	.648	**-0.14**	**-3.96**	**< .001**	**0.22**	**6.27**	**< .001**
Neg. affect	Social excl.	0.00	**-0.08**	**-2.04**	**.041**	0.07	1.70	.089	**-0.13**	**-3.02**	**.003**	**0.32**	**7.12**	**< .001**
Time-Lagged Models														
Paranoia	Social excl.	**0.13**	**0.12**	**2.65**	**.008**	0.03	0.61	.539	**0.11**	**2.07**	**.039**	-0.09	-1.91	.057
Social excl.	Paranoia	0.05	0.02	0.51	.612	0.05	1.20	.229	-0.03	-0.74	.459	0.05	1.30	.192
Paranoia	Neg. affect	-0.02	-0.06	-1.61	.107	0.04	0.98	.327	0.06	1.47	.141	0.03	0.71	.476
Neg. affect	Paranoia	**0.08**	0.04	1.01	.315	0.05	1.32	.185	**-0.08**	**-2.06**	**.040**	**0.13**	**3.23**	**.001**
Social excl.	Neg. affect	0.05	0.01	0.35	.729	0.03	0.83	.406	-0.02	-0.41	.684	0.07	1.82	.068
Neg. affect	Social excl.	0.07	0.06	1.31	.190	0.00	-0.10	.920	**0.13**	**2.58**	**.010**	-0.01	-0.24	.809

*Note.* Neg. affect = negative affect; Social excl. = social exclusion. Models are random-intercept, fixed slope multilevel-regressions with predictor and discrimination index as well as, grand-mean centered negative other-beliefs scores, negative self-beliefs scores, social defeat and the listed interaction effects as independent variables. Time-lagged associations are additionally controlled for the effect of the dependent variable scores from the previous assessment.

^a^*p*-values calculated with Satterthwaite approximation, with significant effects (*p* < .05) printed in bold.

## Discussion

In this study, we aimed to explore whether the extent of intersectional lifetime discrimination experience (LDE) continuously heightens susceptibility to feelings of paranoia/mistrust in minoritized groups. Additionally, we investigated whether this heightened sensitivity can be fully explained by key factors identified in socio-cognitive etiological models of clinical paranoia. In line with previous empirical and theoretical models (Bentall et al., 2014), we found that among all types of psychotic (positive, negative, and depressive) symptoms, paranoia shows the highest association with discrimination. Additionally, LDE is correlated with habitual feelings of social exclusion (i.e., social defeat), one’s negative self-beliefs, and negative beliefs about others. This replicates the established link between LDE and both paranoia and socio-cognitive factors at the core of clinical paranoia models (Freeman et al., 2002; Selten et al., 2013).

When investigating the role of LDE on paranoia in daily life, we consistently found that an increasing complexity of intersectional LDE amplifies the dynamic interrelations of negative affect, state-exclusion, and state-paranoia. Among these, the largest amplifying effects were found for negative affect and state-exclusion on state-paranoia, with the effects for state-exclusion on state-paranoia being found in cross-sectional and time-lagged analyses. These directional effects mirror well-established triggers that have been shown to elicit paranoia both in lab studies (Lamster et al., 2017; Lincoln et al., 2018; Stewart et al., 2017) and numerous ambulatory assessment studies (e.g., Bell et al., 2023). With LDE intensifying this trigger-response-association at the heart of various vulnerability-stress models of paranoia and psychosis (negative emotions, e.g., Freeman et al., 2002; Myin-Germeys & Van Os, 2007; adversity, e.g., Selten et al., 2013), these findings at first glance indicate that discrimination indeed purely constitutes a clinical risk factor.

However, within the healthy cultural mistrust framework, increased mistrust in response to situational stressors could also be found in minoritized groups as an adaptive response to increased exploitation and discrimination (Whaley, 2001b): A person with more LDE might respond with mistrust to social stressors, but this response may not necessarily fall within the spectrum of clinically relevant paranoia and thus would not be governed by the same set of social-cognitive risk factors for clinical paranoia. Consequently, finding moderation effects of LDE that still exist when negative beliefs about oneself, others, and habitual social defeat are controlled for can be interpreted as a sign of non-clinical sensitization processes. Especially the stable moderation effect LDE found on state social exclusion and concurrent/subsequent state-paranoia shows that the differential reaction to current social adversity in people with LDE cannot be fully explained by changes in beliefs and/or habitual feelings of social defeat. This specificity to the association with a current social stressor tentatively suggests that the effects we found could be partially caused by healthy cultural mistrust.

Nevertheless, the reduced effect size of moderating effects of LDE when clinical risk factors were controlled for indicate that at least some of these influences can be explained within a clinical framework. In sum, this everyday-life perspective offers a potential explanation for previous findings from cross-sectional studies that showed a lowered associations between paranoia levels and some, but not all socio-cognitive risk factor levels in intersectionally minoritized groups (Kingston et al., 2023): Based on our results, we could assume that some seemingly-paranoid responses of healthy mistrust emerge in response to situational factors of the eliciting social-exclusion experience, i.e., an increased tendency towards facing new situations of discrimination; and these responses are not rooted in a cognitively biased perception of the situation due to dysfunctional core-schemas. In consequence, retrospective reports of core schemas about oneself and others and paranoid beliefs would be less strong/ubiquitous in minoritized groups due to confounding clinical paranoia and healthy mistrust. To further verify this hypothesis, future research needs to provide a more detailed picture of the nature of the everyday-life situations that elicit feelings of social exclusion in minoritized people and elucidate whether discriminatory features of these situational triggers contribute to an increase in paranoid responses when compared to non-minoritized groups.

This is, to our knowledge, the first attempt at disentangling the clinical spectrum of paranoia and healthy mistrust using ambulatory assessment and a competing moderation approach. Given that multiple clinical models exist besides the social and cognitive ones included here (for an overview, see Denecke et al., 2024), we can only preliminarily draw the conclusion that the moderating effects of LDE are outside the working mechanisms of clinical models of paranoia. It might well be that other mechanisms described in clinical models, for example, reasoning biases as recently framed within Bayesian inference paradigms (Coltheart et al., 2010; Fletcher & Frith, 2009), fully account for the LDE moderation effect we found. Even if that were to be the case, however, it would lead to theoretically and practically important follow-up questions of where to draw the line and declare a response of mistrust as clinical paranoia, and which associated mechanisms are crucial to this distinction.

Finally, some general limitations need to be considered: First, our sample is a population sample. While previous work has demonstrated multiple times that the spectrum of paranoia extends to the general population (Elahi et al., 2017), a comparison of clinical groups with and without minority backgrounds would provide more substantial evidence that the differences we found persist when treatment-relevant levels of distress are reached. Also, our study constituted a secondary analysis of existing data, so a pre-registered verification of our findings (preferably in population and clinical samples) is needed. Second, the vast majority of the healthy cultural mistrust research revolved around ethnicity. Some recent studies (Ellett et al., 2025; Kingston et al., 2023) provided evidence that this type of research can be generalized to other minority groups and other forms of discrimination, but with our comparatively small subsamples for individual minorities, we are unable to adequately analyse and compare different types of discrimination individually. Follow-up studies may benefit from closing this gap between our research and the bulk of healthy mistrust studies by stratifying participants along one or multiple types of minority group association. Finally, lifetime discrimination has been based on a broadly defined self-report assessment in this study. Consequently, certain degrees of nuance in intersectional minority status or discrimination could not be adequately captured (e.g., differentiation between gender-based discrimination and transphobic discrimination or discrimination because of sexual orientation vs. because of sexual orientation and sexual identity). This degree of imprecision could have led us to systematically underestimate associations involving complex intersectional discrimination. Further, relying on self-report leads to a risk of confounding an accurate assessment of discrimination with a bias due to paranoid thinking, possibly leading to an inflation of reported discrimination. While the complexity of our study, specifically the separation of state-paranoia and state-triggers in ambulatory assessment and discrimination as part of the baseline questionnaire as well as the pattern of our results showing associations between discrimination and subsequent situational dynamics of state-paranoia can be seen as arguments against this risk playing a prominent role, a more extensive assessment of discrimination experience (e.g., a interview-based assessment of factual descriptions of the situations perceived as discriminatory or a third person assessment via participants relatives or close friends) could provide a more valid measure of this variable in future research. Similarly, the baseline assessment of chronic social exclusion/social defeat was rather brief. While some aspects of chronic social defeat commonly used as additional indicators are derived from self-reported discrimination experiences and cannot be implemented as a social defeat measure in a study that aims to disentangle minoritization effects from social defeat, future studies could expand the assessment of chronic defeat by including additional constructs (e.g., self-reported lifetime feelings of ostracism; Shovestul et al., 2023) to increase the quality of chronic social defeat assessment.

In conclusion, our research shows that a history of (intersecting) discrimination experiences increases state-mistrust in response to (social) stress and that psychosocial clinical models of paranoia cannot fully explain this effect. Thus, healthy cultural mistrust could partially explain such reactions that are mistakenly labelled as paranoid ideation. By incorporating this perspective, we can further refine our understanding of paranoia and, through future research, optimize evidence-based guidelines for diversity-sensitive treatment.

## Supplementary Materials

The Supplementary Materials contain an overview of the full ambulatory assessment questionnaire (for access, see Schlier et al., 2026S).



SchlierB.
StrakeljahnF.
KahlC.
WinklerK.
 (2026S). Supplementary materials to "Are you really paranoid, just because they are after you? Exploring the underlying sensitization processes of intersectional discrimination on everyday-life paranoia"
[Overview of the full ambulatory assessment questionnaire]. PsychOpen. 10.23668/psycharchives.21893


## Data Availability

Data supporting the conclusions of this study are available upon reasonable request to the corresponding author. Data are not publicly available due to lack of explicit consent to open data publication in the informed consent.

## Preregistration

This study is not pre-registered.

## Reporting Guidelines

The JARS-Quant guidelines for non-experimental quantitative research were followed in this study.
